# Brugada-type Electrocardiographic Pattern Induced by Fever

**Published:** 2005-04-01

**Authors:** Ozcan Ozeke, Dursun Aras, Bilal Geyik, Bulent Deveci, Timur Selcuk

**Affiliations:** Yuksek Ihtisas Hospital, Department of Cardiology, Ankara, Turkey

**Keywords:** Brugada syndrome, Fever

## Abstract

ST-segment elevation in Brugada syndrome is caused by a shift in the ionic current balance and the creation of a voltage gradient between the epicardium and the endocardium. This ionic mechanism have been shown to be temperature dependent. We describe a 33-year-old man who presented with fever with the dynamic electrocardiographic changes similar to the Brugada syndrome. These electrocardiographic anomalies disappeared when the temperature returned to normal.

## Case Report

**Figure 1:**
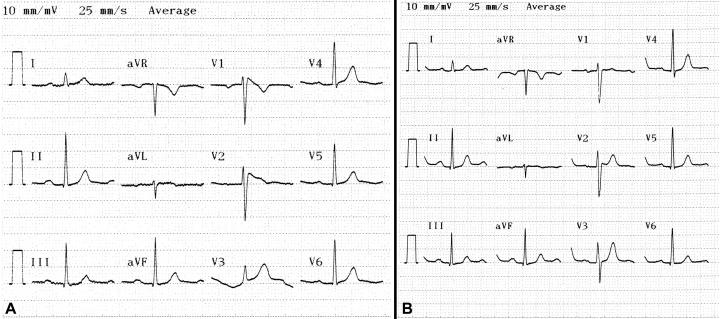
12-lead electrocardiogram shows incomplete right bundle branch block with coved type ST-segment elevation in leads V1-V3, and first-degree atrioventricular block at the condition of fever (1A) and shows minimal ST-segment elevation in V2 without the incomplete right bundle branch block at the rest without fever (1B).

**Figure 2:**
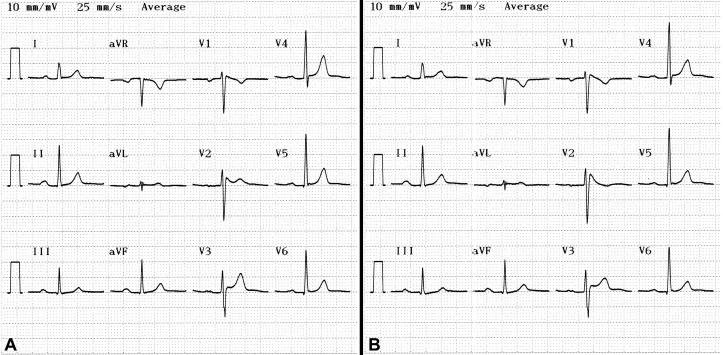
12-lead electrocardiogram shows that the ST-segment elevation changed from the saddle-back type (2A) to the coved type (2B) with drug challenge.

A 33-year-old man with fever due to common cold was referred to our hospital with suspicion of having an acute coronary syndrome. Three hours before admission, the patient with complaints of fever, chills, sore throat, was admitted to a local hospital, where the temperature was 38.7°C and his electrocardiogram showed characteristic Brugada pattern with coved type ST-segment elevation in leads V1-V3 and first-degree atrioventricular block (Figure 1). He was diagnosed as having common cold and was referred to our hospital because of his electrocardiographic changes resembling acute coronary syndrome. He had no cardiac complaints and did not have a history of syncope or a family history of sudden cardiac death. On physical examination, the patient was dehydrated but alert. In the emergency room, his temperature was 38.3°C, the blood pressure was 120/70 mm Hg and the pulse was 88 per min. There was an inflammation of the pharynx. No other abnormalities were found. Hematological and biochemical tests including cardiac enzymes and electrolytes were within normal limits and the chest X-ray was unremarkable.The white blood cell count was 9600/cu mm. There was no evidence of structural heart disease on echocardiographic examination. Later that day, the patient’s temperature was 36.9°C  with  antipyretics and electrocardiogram showed saddle-back type ST-segment elevation in leads V1-V2 (Figure 2A). Subsequently, drug challenge test with propafenone was performed; the ST-segment elevation changed from the saddle-back type (Figure 2A) to the coved type (Figure 2B). The next day, the patient was afebrile and the incomplete right bundle branch block disappeared on electrocardiogram, which showed only minimal ST-segment elevation in V2 (Figure 1B). An electrophysilogical study was not performed because neither the patient nor any of his family members had experienced any arrhytmic symptom or sudden cardiac death, and medical follow up was decided. The patient was recommended to came hospital for urgent therapy of fever when a fever developed.

## Discussion

Brugada syndrome, first described as a new clinical entity by Pedro and Josep Brugada in 1992, is an inherited cardiac disease causing life-threatening ventricular tachyarrhythmias in patients with structurally normal heart and a characteristic electrocardiogram showing a pattern of right bundle branch block and ST segment elevation in right precordial leads V1 to V3 [1]. Wilde et al [2] reported 3 types of repolarization patterns as the ECG criteria for diagnosis of Brugada Syndrome; type 1 ECG, a coved-type ST segment elevation of ≥ 2 mm; type 2 ECG, saddle-back type a J wave amplitude of ≥ 2 mm and gradually descending ST segment elevation remaining ≥ 1 mm above baseline; and type 3 ECG, a coved-type or saddle-back type with ST segment elevation of ≤ 2 mm.

In spite of all the knowledge acquired over the last decade on this syndrome, diagnosis and risk stratification can be difficult sometimes. The ECG pattern may be dynamic over time and may include transient normalization [2]. The pathophysiology of Brugada syndrome is still under investigation. Insight from cellular electrophysiology suggests that the ST-segment elevation is caused by a shift in the ionic current balance and the creation of a voltage gradient, with predominance of transient outward current in the epicardium over the endocardium [3]. The ionic mechanism responsible for the Brugada syndrome have been shown to be temperature dependent [4-8]. In the present case, the electrocardiographic anomalies were evident when the fever was present and disappeared once the temperature returned to normal. It is possible that an increase in temperature may be the most important factor in the appearance of these electrocardiographic changes. In consideration of its variability, physicians should be aware of its often transient electrocardiographic features and fever should be aggressively treated. Our present report supports the effect of the body temperature in diagnosis and of on the underlying ion channelopathy of Brugada syndrome.
